# Redo laparoscopic pyeloplasty for recurrent ureteropelvic junction obstruction: Propensity score matched analyses of a high-volume center

**DOI:** 10.3389/fped.2022.997196

**Published:** 2022-09-08

**Authors:** Jiayi Li, Yang Yang, Zonghan Li, Songqiao Fan, Xinyu Wang, Zhenzhen Yang, Pei Liu, Hongcheng Song, Weiping Zhang

**Affiliations:** Department of Urology, National Center for Children's Health, Beijing Children's Hospital, Capital Medical University, Beijing, China

**Keywords:** ureteropelvic junction obstruction, redo laparoscopic pyeloplasty, primary laparoscopic pyeloplasty, redo open pyeloplasty, children

## Abstract

**Purpose:**

Review the experience of redo laparoscopic pyeloplasty (RLP) in patients with recurrent ureteropelvic junction obstruction (UPJO) in comparison to primary laparoscopic pyeloplasty (PLP) and redo open pyeloplasty (ROP), and determine the feasibility and effectiveness of RLP for recurrent UPJO in children.

**Methods:**

We retrospectively reviewed the clinical data of patients treated with transperitoneal PLP, RLP, and ROP for UPJO from December 2015 to December 2022. The Propensity score matching (PSM) was used to balance confounding variables. RLP patients were 1:4 matched with PLP and 1:3 matched with ROP. The primary outcomes were failure and post-operative complications. Complications were classified according to the Clavien-Dindo grading system.

**Results:**

The study included ten patients who underwent RLP, 43 patients who underwent ROP, and 412 patients who underwent PLP. The follow-up time ranged from 6 to 36 months in the RLP group, 12 to 60 months in the PLP group, and 24 to 54 months in the ROP group. In the RLP group, no failure but three post-operative complications (Clavien grade II) were observed during the follow-up. Compared with the PLP group, the older age, higher weight, larger pre-operative anteroposterior diameter (APD) and APD/cortical thickness (P/C ratio), longer operation time, and post-operative length of stay (LOS) in the RLP group (*P* < 0.05). After PSM, longer operation time and post-operative LOS were observed in the RLP group (*P* < 0.05). Compared with the ROP group, the older age, higher weight, and longer post-operative LOS in the RLP group (*P* < 0.05). After PSM, longer post-operative LOS was observed in the ROP group (*P* < 0.05). The failure and complication rates were comparable between RLP and PLP or RLP and ROP (*P* > 0.05).

**Conclusions:**

Our result demonstrated that RLP performed as well as PLP except for a longer operation time. Compared with ROP, RLP has the advantages of a clearer surgical view, sufficient exposure, clearer anatomical landmark position, and minor trauma with a comparable clinical outcome. On experienced hands, RLP for recurrent UPJO after is a safe and effective procedure and should be considered an excellent alternative to the more commonly recommended ROP in select patients.

## Introduction

Ureteropelvic junction obstruction (UPJO) is the most common cause of hydronephrosis in children, and the Andersone-Hynes dismembered pyeloplasty has been the gold standard repair (1). For decades, with the widespread adoption of minimally invasive techniques as alternatives to open surgery, laparoscopic pyeloplasty (LP) has been widely accepted, which benefits from decreasing operative times, blood loss, analgesic requirements and post-operative stays compared with open pyeloplasty (OP) (2). Compared with primary pyeloplasty, the redo pyeloplasty is an operative challenge due to the expected scarring and altered anatomic planes. With the fibrosis and distorted anatomy of the recurrent UPJO, redo open pyeloplasty (ROP) has traditionally been viewed as the reference standard for many urologists (3, 4). Recently, small case series studies have revealed the feasibility and effectiveness of redo laparoscopic pyeloplasty (RLP) in infants and children (5, 6). Nonetheless, limited studies directly compare the outcomes of RLP with primary laparoscopic pyeloplasty (PLP) and ROP.

In the present study, we reported our outcomes of recurrent UPJO patients treated with RLP in a high-volume center and compared these patients with patients who underwent PLP and ROP. The Propensity score matching (PSM) method was implemented to construct to balance confounding variables. We hypothesized that in experienced hands, RLP is feasible and effective for recurrent UPJO, with a comparable clinical outcome compared with PLP and ROP.

## Materials and methods

### Patients

The clinical data of children with primary Andersone-Hynes LP for UPJO and redo pyeloplasty (including RLP and ROP) were retrospectively reviewed and analyzed between December 2016 to December 2022 in the Department of Urology, Beijing Children's Hospital. The exclusion criteria are as follows: 1. Patients with a duplex or solitary kidney. 2. Patients combined with vesicoureteral reflux. 3. Patients with bilateral UPJO. 4. Patients with incomplete data or lost to follow-up.

UPJO was diagnosed based on the patient's symptoms and clinical examinations. Surgical intervention was recommended when a patient had one or more of the following conditions: a. Ultrasonography showed progression of hydronephrosis, b. Patients with symptomatic renal colic, urinary tract infection, and severe upper urinary tract dilatation (Society of Fetal Urology grade III or IV), c. The renal function of the hydronephrotic kidney is <40%. Moreover, the DTPA renogram demonstrated an obstructive pattern (defined as T1/2 > 20 min after administration of furosemide) for reference only. Surgical success was defined as symptom resolution, anteroposterior diameter (APD) decrease, pelvis and caliceal tension decrease in renal ultrasounds, ureters well seen within 40 min in intravenous pyelography or post-operative t1/2 improvement during follow-up (7). Failure was defined as the recurrent UPJO need to redo dismembered pyeloplasty based on a post-operative obstruction, persistent or worsening hydronephrosis, or symptomatic obstruction.

The patient's pre-operative data, intraoperative parameters, and follow-up information were collected. Pre-operative data included patient's age, sex, weight, pre-operative presentation, and ultrasound parameters. For redo pyeloplasty, primary surgery, the interval between primary and redo pyeloplasty and temporizing interventions were also collected. Intraoperative parameters included operation time, operation side, surgeon, and intraoperative drainage. For redo pyeloplasty, restenosis reasons were also collected. Follow-up information mainly included length of stay (LOS) after surgery, post-operative complications, and failure. Ultrasound parameters include APD, cortical thickness, APD/cortical thickness ratio (P/C ratio), and percentage of improvement of APD (PI-APD). Post-operative complications were classified according to the Clavien-Dindo classification (8). The primary outcomes of the present study were failure and post-operative complications.

The study was conducted in accordance with the Declaration of Helsinki (as revised in 2013). This study obtained approval from our institutional ethical review board [IEC-C-006-A04-V.06, (2022)-E-030-R]. Individual consent for this retrospective analysis was waived.

### Surgical techniques

**Figure 1:**
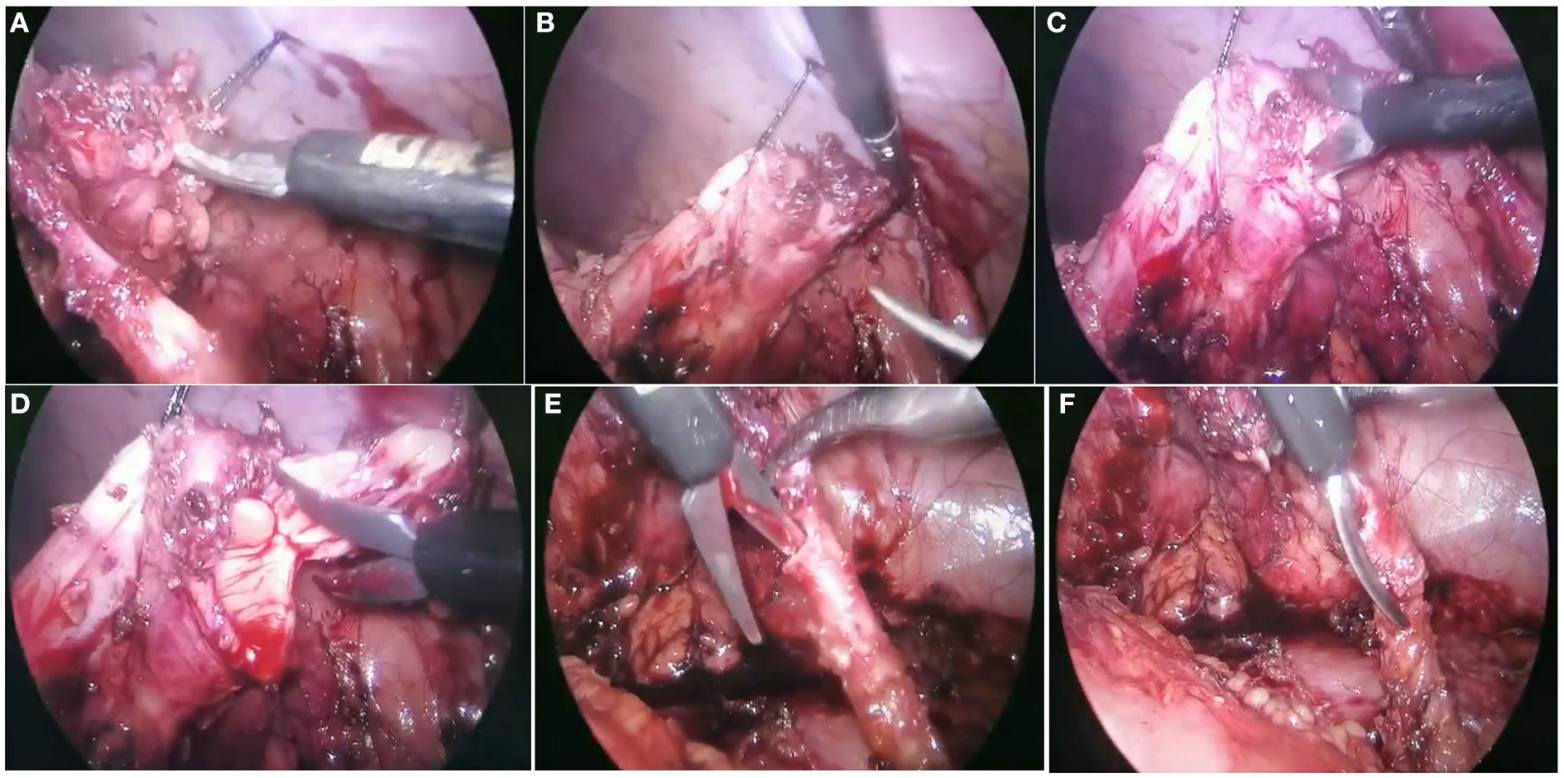
Recurrent ureteropelvic junction obstruction (UPJO) patient treated with redo laparoscopic pyeloplasty (RLP). **(A,B)** Identified the reason for restenosis of the primary pyeloplasty was severe scar hyperplasia around the UPJ area (arrows). The normal ureter was then identified distally and dissection was carried out proximally toward the renal pelvis. **(C,D)** Refashioning the distended renal pelvis with excision of the redundant part. **(E,F)** Excised the fibrotic segment and excised the ureter laterally for about 1 cm.

PLP was performed through a transperitoneal approach with three ports (5 mm). Five to zero absorbable monofilament running suture was used in all cases. The colon was mobilized to expose the renal pelvis after removing all the adhesions until the UPJ was identified. After UPJ was dismembered and the stenotic segment was removed, the double-J (DJ) stent was first tried by surgeons. For those with DJ stent well placed in an antegrade fashion, perinephric drain and urethral catheter were placed simultaneously. The appropriate catheter size was selected based on the patient's age. For Patients aged 0–2, 2–5, 5–10, and 10–16 years, 6, 8, 8–10, and 10–12 Fr catheters were selected, respectively. As for the DJ stent, a 4.7F stent is commonly selected. If the age- and height-appropriate stent was difficult to place and changing to a smaller size still had significant resistance at the ureterovesical junction, this situation was considered as a difficult process of inserting DJ stent, and nephrostomy tube plus external ureteral stent were indwelled as an alternative drainage method. For RLP, after exploring the peritoneal cavity, the renal pelvis was exposed with medial mobilization of the colon regardless of the laterality, and the reason for the restenosis of the previous pyeloplasty was identified. The peripelvic fibrosis was gently released by blunt and sharp dissection. The normal ureter was then identified distally, and dissection was carried out proximally toward the renal pelvis. The UPJ was usually found as a thick fibrotic area connecting the renal pelvis with the rest of the ureter. Then we excised the fibrotic segment and excised the ureter laterally for about 1 cm while refashioning the distended renal pelvis with excision of the redundant part. Intraoperative pictures of RLP are shown in Figure 1. For those patients with prior ureterocalicostomy, the DJ sent was indwelled while the nephrostomy tube was retained. The ROP technique follows the same steps as the RLP, and the nephrostomy tube and external ureteral stent were indwelled as the routine drainage method. All procedures were performed by surgeons with the same qualifications of pyeloplasty surgery. Surgeons were classified into chief physician and associate chief physician groups based on their experience.

Post-operatively, oral feeding is given once the patient experiences flatulence, defecation, or reappearance of bowel sounds. The perinephric drain was removed when the remaining output of the drainage increased <10 ml within 24 h. The Foley catheter and external ureteral stent were removed before the patient's discharge. After discharge, prophylactic antibiotic (cephalosporin, 50 mg/kg. d) was maintained for 1–2 weeks. The nephrostomy tube was removed in accordance with the methylene blue study before discharge, which was usually 10–14 days after surgery, and cystoscopic removal of the DJ stent was done under general anesthesia at 4–6 weeks post-operatively. Patients with both a DJ sent and a nephrostomy tube (patients with prior ureterocalicostomy before RLP) were discharged with nephrostomy tube and DJ sent after RLP surgery. After DJ sent removal, the nephrostomy tube was removed after the assessment of patency of UPJ and ureter. Routine follow-up for all patients included assessment in the clinic at 3 (after DJ sents removal), 6, 12, 18, and 24 months post-operatively under outpatient review or telephone interview.

### Statistical analysis

The propensity score matching (PSM) method was implemented to construct paired matched samples of two groups to balance confounding variables. A propensity score for each patient was calculated using the multivariable logistic regression model. The cases were matched to the nearest-neighbor controls based on their propensity scores. RLP patients were 1:4 matched with PLP patients and 1:3 matched with ROP patients.

Statistical analysis was conducted by R software (version 4.0.3, http://www.r-project.org). Continuous data not following normal distribution are presented as median and inter-quartile range, analyzed by Mann-Whitney *U*-test, while variables between groups were compared using Chi-square test or Fisher exact test. All statistical results were reported as two-tailed *P* < 0.05 is considered statistical significance.

## Results

A total of 504 patients underwent PLP, and 61 patients underwent redo pyeloplasty (11 underwent RLP and 50 underwent ROP) were incorporated in the study. One hundred patients were excluded according to the exclusion criteria: Ten patients with duplex kidney or solitary kidney, seven patients combined with vesicoureteral reflux, 17 patients with bilateral UPJO, and 66 patients with incomplete data or lost to follow-up. Finally, 412 patients underwent PLP, ten patients underwent RLP, and 43 underwent ROP were further included in the study.

### Characteristics and outcomes of RLP patients

Ten patients (seven boys and three girls) underwent RLP for recurrent UPJO, of which seven patients (70.0%) were referred from other hospitals after failed pyeloplasty. The median age for RLP surgery was 91.1 [49.8; 114] months, and the median weight was 22.6 [16.9; 36.2] kg. All patients were Society for Fetal Urology (SFU) grade IV. Four patients (40.0%) presented initially with flank pain, nausea or vomiting, and associated hydronephrosis. Seven patients (70.0%) had previously undergone LP, and three (30.0%) had previously undergone OP. Before the RLP, two patients underwent ureterocalicostomy, and one patient underwent endopyelotomy as temporizing interventions. The interval between primary and redo pyeloplasty was 7.50 [6.25; 14.8] months. The median operative time of RLP was 158 [120; 197] min, without conversion to open surgery. During the operation, the reason for restenosis of the primary pyeloplasty was identified, including five patients (50.0%) developed severe scar hyperplasia around the UPJ area, adhesions causing obstruction were observed in two patients (20.0%), stenotic UPJ area was observed in one patient (10%), crossing vessels was observed in one patient (10.0%), UPJ polyps was observed in one patient (10%). Three patients (30.0%) difficult to insert a DJ stent during the operation, and a nephrostomy tube plus an external ureteral stent were indwelled as the alternative drainage method. No other intraoperative complication was observed. Post-operatively, one patient had intestinal paralysis resolved after fasting and decompression with a gastrointestinal tube (Clavien II). Median LOS after surgery was 6 [5.25; 7.75] days. Four patients prolonged the LOS due to the indwelling nephrostomy tube plus an external ureteral stent during the surgery. Two patients developed febrile urinary tract infection (UTI) after DJ stent removal and were relieved after receiving antibiotics therapy (Clavien II). The post-operative complication rate of the RLP group was 30.0% (as shown in Table 1). The follow-up duration was 6 to 36 months, and all patients demonstrated an improvement in hydronephrosis without failure. Ultrasound parameters demonstrated that the post-operative APD is significantly smaller than pre-operative APD [2.20 (1.27; 3.50) vs. 4.45 (3.75; 5.92) cm, *P* < 0.01], and the post-operative P/C ratio is significantly lower than pre-operative P/C ratio [4.67 (3.04; 7.04) vs. 15.8 (8.06; 20.3), *P* < 0.01]. The post-operative cortical is thicker than the pre-operative cortical, but the difference was not statistically significant [0.40 (0.29; 0.65) vs. 0.30 (0.21; 0.40) cm, *P* = 0.06]. The PI-APD was 0.63 ± 0.22 during the follow-up.

**Table 1:** Complications after primary and redo pyeloplasty.

**Complications**	**Clavien Dindo grading**	**RLP, *n* (%)**	**PLP, *n* (%)**	**ROP, *n* (%)**
febrile UTI	II	2 (20.0%)	30 (7.28%)	4 (9.30%)
Intestinal paralysis	II	1 (10.0%)	2 (0.49%)	1 (2.33%)
Ileus	IIIb	/	/	1 (2.33%)
UPJ leakage	IIIb	/	1 (0.24%)	/
Hernia formation	IIIb	/	4 (0.97%)	/
Prolapse of nephrostomy tube	IIIb	/	1 (0.24%)	/
Delayed wound healing	IIIb	/	2 (0.49%)	1 (2.33%)
Infected allantois formation	IIIb	/	1 (0.24%)	/
DJ stent migration	IIIb	/	1 (0.24%)	/
Distal ureteral stricture	IIIb	/	4 (0.97%)	/
Recurrunt UPJO	IIIb	/	12 (2.91%)	2 (4.65%)

PLP, primary laparoscopic pyeloplasty, RLP, redo laparoscopic pyeloplasty, ROP, redo open pyeloplasty, UTI, urinary tract infection, UPJ, ureteropelvic junction, DJ, Double-J, UPJO, ureteropelvic junction obstruction.

### Comparison of RLP vs. PLP

As shown in Table 2, 338 (82.0%) boys and 74 (18.0%) girls are in the PLP group. The median age for PLP surgery was 48.9 [20.3; 90.4] months, and the median weight in the PLP group was 17.0 [11.9;26.0] kg, which is significantly lower than the RLP group (*P* < 0.05). The pre-operative APD in the PLP group was smaller than that in the RLP group [2.90 (2.20; 3.70) vs. 4.45 (3.75; 5.92) cm, *P* = 0.009], and the pre-operative P/C ratio is significantly lower than RLP group [15.8 (8.06; 20.3) vs. 7.67 (4.50; 14.0), *P* < 0.01]. The median operative time of PLP was 110 [84.0; 140] min, which is significantly shorter than the RLP group (*P* = 0.015). Post-operative LOS after surgery in the PLP group was 5 [5.00; 7.00] days, shorter than that in the RLP group (*P* = 0.018). The follow-up period of the PLP group ranged from 14.2 to 78 months. As shown in Table 1, 58 patients (14.1%) developed complications after PLP (Table 1), of which 30 patients developed febrile UTI after DJ stent or nephrostomy tube removal and relieved after receiving antibiotics therapy (Clavien II), two developed intestinal paralyzes and relieved after fasting plus gastrointestinal decompression. Besides, three patients underwent debridement and suturing because of hernia formation (Clavien IIIb), and one underwent hematoma evacuation because of intra-abdominal bleeding and hematoma formation (Clavien IIIb). Prolapse of the nephrostomy tube occurred in one patient, and he underwent the nephrostomy (Clavien IIIb). One patient exhibited poor wound healing around the fistula and received fistula extraction, debridement and reclosure (Clavien IIIb). Infected allantois formation was observed in one patient, he underwent the nephrostomy (Clavien IIIb). DJ stent migration occurred in one patient, we reset the DJ stent (Clavien IIIb). The distal ureteral stricture was observed in 4 patients, they underwent ureteral reimplantation after PLP (Clavien IIIb). Recurrent UPJO (Failure) was observed in 12 patients (2.91%) during the follow-up, they underwent redo pyeloplasty. The post-operative complication and failure rates were insignificant between the PLP and RLP groups (*P* > 0.05).

**Table 2:** Characteristics for patients underwent primary and redo laparoscopic pyeloplasty.

	**Before PSM**			**After PSM**		
	**RLP (*n* = 10)**	**PLP (*n* = 412)**	***P*-value**	**RLP (*n* = 9)**	**PLP (*n* = 31)**	***P*-value**
Sex (M/F)	7/3	338/74	0.399	6/3	27/4	0.319
Age (months)	91.1 [49.8; 114]	48.9 [20.3; 90.4]	**0.040**	96.1 [45.0; 119]	86.2 [22.1; 116]	0.616
Weight (kg)	22.6 [16.9; 36.2]	17.0 [11.9; 26.0]	**0.042**	23.7 [16.0; 37.0]	24.5 [13.2; 41.0]	0.697
Kidney malformation (Y/N)	0/10	7/405	1.000	0/9	2/29	1.000
Presentation (Y/N)	4/6	174/238	1.000	3/6	15/16	0.476
Side (L/R)	7/3	87/325	0.450	6/3	27/4	0.316
Pre-operative APD (cm)	4.45 [3.75; 5.92]	2.90 [2.20; 3.70]	**0.009**	4.10 [3.70; 5.70]	3.50 [2.65; 5.50]	0.549
Pre-operative cortical thickness (cm)	0.30 [0.21; 0.40]	0.40 [0.20; 0.50]	0.270	0.30 [0.20; 0.40]	0.30 [0.22; 0.45]	0.42
Pre-operative P/C ratio	15.8 [8.06; 20.3]	7.67 [4.50; 14.0]	**0.035**	13.0 [7.33; 19.0]	8.80 [6.50; 24.2]	0.6
Post-operative APD (cm)	2.20 [1.27; 3.50]	1.60 [1.20; 2.10]	0.256	2.40 [1.15; 3.60]	1.70 [1.30; 3.15]	0.97
Post-operative cortical thickness (cm)	0.40 [0.29; 0.65]	0.40 [0.30; 0.60]	0.541	0.35 [0.29; 0.55]	0.40 [0.30; 0.60]	0.699
Post-operative P/C ratio	4.67 [3.04; 7.04]	3.29 [2.18; 5.75]	0.357	5.12 [3.58; 7.77]	4.49 [2.12; 10.1]	0.915
Operation time (min)	158 [120; 197]	110 [84.0; 140]	**0.015**	166 [135; 203]	110 [87.5; 132]	**0.006**
Surgeon (Chief/Associate chief)	5/5	194/218	1.000	5/4	16/15	1.000
Post-operative LOS (d)	6.00 [5.25; 7.75]	5.00 [5.00; 6.00]	**0.018**	6.00 [6.00; 8.00]	5.00 [5.00; 6.00]	**0.037**
Complications (Y/N)	3/7	58/354	0.164	3/6	11/20	1.000
Failure (Y/N)	0/10	12/400	1.000	0/9	1/30	1.000

PLP, primary laparoscopic pyeloplasty; RLP, redo laparoscopic pyeloplasty; APD, anteroposterior diameter; P/C ratio, APD/cortical thickness; LOS, length of stay.

Bold values indicate statistical significance.

To eliminate the patient-dependent bias, the 1:4 matched PSM method was generated with a caliper distance of 0.10. After PSM, nine patients in the RLP group and 39 patients in the PLP group were matched. Patients' characteristics including age, weight, surgeon experience, pre-operative APD, cortical thickness, and P/C ratio were all balanced (*P* > 0.05). After PSM, the operative time of PLP and post-operative LOS after surgery in the PLP group was still shorter than that in the RLP group (*P* = 0.018). The serious complication and failure rates were still insignificant between the PLP and RLP groups (*P* > 0.05).

### Comparison of RLP vs. ROP

The clinical characteristics of RLP and ROP patients are summarized in Table 3. There are 40 (93.0%) boys and three (7.0%) girls in the ROP group. The median age at ROP surgery was 48.1 [26.6; 77.3] months, and the median weight in the ROP group was 17.5 [11.6; 21.5] kg, which is significantly lower than that in the RLP group (*P* < 0.05). In the ROP group, 24 patients (55.8%) had previously undergone LP, and 19 (44.2%) had previously undergone OP. The interval between primary and redo pyeloplasty was 10.0 [8.00; 15.5] months in the ROP group. Before the ROP, nine patients underwent ureterocalicostomy, and six underwent endopyelotomy as temporizing interventions. The pre-operative APD in the ROP group was 5.40 ± 1.10 cm, the pre-operative cortical thickness was 0.20 [0.10; 0.30] cm, pre-operative P/C ratio was 31.5 [12.8; 53.5], which is not significantly different from those in the RLP group (*P* > 0.05). The median operative time of ROP was 127 [87.0; 160] min, shorter than that in the RLP group, but the difference was insignificant (*P* = 0.176). Post-operative LOS after surgery in the ROP group was 11 [9.00; 13.00] days, longer than that in the RLP group (*P* < 0.001). The follow-up period of the ROP group ranged from 15 to 79 months. In the ROP group, nine patients (20.9%) developed post-operative complications (as shown in Table 1), of which four patients developed febrile UTI after nephrostomy tube removal and relieved after receiving antibiotics therapy (Clavien II), one developed intestinal paralysis and relieved after fasting plus gastrointestinal decompression. One patient of ileus with ineffective conservative treatment underwent exploratory laparotomy plus bowel adhesiolysis (Clavien IIIb). One patient exhibited poor wound healing around the fistula and received fistula extraction, debridement and reclosure (Clavien IIIb). Recurrent UPJO (Failure) occurred in two patients (4.65%), they underwent redo pyeloplasty. The complication and failure rates were insignificant between the ROP and RLP groups (*P* > 0.05).

**Table 3:** Characteristics for patients underwent redo laparoscopic pyeloplasty and redo open pyeloplasty.

	**Before PSM**			**After PSM**		
	**RLP (*n* = 10)**	**ROP (*n* = 43)**	***P*-value**	**RLP (*n* = 10)**	**ROP (*n* = 25)**	***P*-value**
Sex (M/F)	7/3	40/3	0.073	7/3	23/2	0.128
Age (months)	91.1 [49.8; 114]	48.1 [26.6; 77.3]	**0.045**	91.1 [49.8; 114]	60.6 [43.6; 80.8]	0.189
Weight (kg)	22.6 [16.9; 36.2]	17.5 [11.6; 21.5]	**0.034**	22.6 [16.9; 36.2]	19.0 [15.0; 23.0]	0.160
Kidney malformation (Y/N)	0/10	4/39	1.000	0/10	2/23	1.000
Presentation (Y/N)	4/6	24/19	0.488	4/6	11/14	1.000
Side (L/R)	7/3	31/12	1.000	7/3	19/6	0.694
Primary surgery (LP/OP)	7/3	24/19	0.494	7/3	14/11	0.704
Interval between primary and redo pyeloplasty (months)	7.50 [6.25; 14.8]	10.0 [8.00; 15.5]	0.269	7.50 [6.25; 14.8]	12.0 [8.00; 19.0]	0.128
Temporizing interventions			1.000			0.92
None	6 (60.0%)	24 (55.8%)		6 (60.0%)	17 (68.0%)	
Ureterocalicostomy	2 (20.0%)	9 (20.9%)		2 (20.0%)	4 (16.0%)	
Endopyelotomy	1 (10.0%)	6 (14.0%)		1 (10.0%)	3 (12.0%)	
Others	1 (10.0%)	4 (9.30%)		1 (10.0%)	1 (4.00%)	
Pre-operative APD (cm)	4.45 [3.75; 5.92]	5.40 (1.10)	0.295	4.45 [3.75; 5.92]	5.36 (0.94)	0.322
Pre-operative cortical thickness (cm)	0.30 [0.21; 0.40]	0.20 [0.10; 0.30]	0.063	0.30 [0.21; 0.40]	0.20 [0.10; 0.40]	0.441
Pre-operative P/C ratio	15.8 [8.06; 20.3]	31.5 [12.8; 53.5]	0.063	15.8 [8.06; 20.3]	25.0 [10.0; 48.0]	0.245
Post-operative APD (cm)	2.20[1.27; 3.50]	2.12 (0.98)	0.724	2.20 [1.27; 3.50]	2.25 [1.30; 3.12]	0.862
Post-operative cortical thickness (cm)	0.40 [0.29; 0.65]	0.40 [0.30; 0.50]	0.716	0.40 [0.29; 0.65]	0.45 (0.23)	0.958
Post-operative P/C ratio	4.67 [3.04; 7.04]	5.00 [3.69; 8.19]	0.654	4.67 [3.04; 7.04]	4.96 [3.44; 8.44]	0.721
PI-APD	0.63 (0.22)	0.58 (0.18)	0.624	0.63 (0.22)	0.58 (0.18)	0.626
Operation time (min)	158 [120; 197]	127 [87.0; 160]	0.176	158 [120; 197]	133 (62.4)	0.244
Surgeon (Chief/Associate chief)	5/5	26/17	0.724	5/5	15/10	0.712
Intraoperative drainage			**<0.001**			**<0.001**
Double-J sent	7 (70.0%)	1 (2.33%)		7 (70.0%)	0 (0.00%)	
Nephrostomy tube	3 (30.0%)	42 (97.7%)		3 (30.0%)	25 (100%)	
Post-operative LOS (d)	6.00 [5.25; 7.75]	11.0 [9.00; 13.0]	**0.001**	6.00 [5.25; 7.75]	10.0 [8.00; 12.0]	**0.003**
Complications (Y/N)	3/7	9/34	0.677	3/7	5/20	0.661
Failure (Y/N)	0/10	2/41	1.000	0/10	1/24	1.000

RLP, redo laparoscopic pyeloplasty, ROP, redo open pyeloplasty, APD, anteroposterior diameter, P/C ratio, APD/cortical thickness, PI-APD, percentage of improvement of APD, LOS, length of stay.

Bold values indicate statistical significance.

The 1:3 matched PSM method was generated with a caliper distance of 0.10. After PSM, ten patients in the RLP group and 25 in the PLP group were matched. Patients' age and weights were balanced (*P* > 0.05). After PSM, the post-operative LOS after surgery in the ROP group was longer than that in the RLP group (*P* < 0.001). The post-operative complication and failure rates were still insignificant between the ROP and RLP groups (*P* > 0.05).

## Discussion

Recurrent UPJO is one of the main complications after primary pyeloplasty, which may occur following primary pyeloplasty in up to 11% of patients who may require redo surgical intervention (9). Compared with primary pyeloplasty, the redo pyeloplasty is challenging, and the success rates are lower than that of the primary surgery (10). Previous studies revealed that (9) the success rate of primary pyeloplasty might be overestimated due to the insufficient follow-up time, and some patients may experience recurrent obstruction for a longer period after surgery and require re-intervention (9). Recurrent UPJO can cause pain, recurrent UTI, fever, and progressive impairment of renal function. Once the obstruction reemerges after the primary pyeloplasty, it is necessary to identify and take interventions as soon as possible to protect the fragile renal function. Several measures for treating recurrent UPJO include DJ stent placement, endopyelotomy, balloon dilation, and redo pyeloplasty (4, 11). Endopyelotomy has the advantages of minimally invasive, short hospital stay, and quick recovery. However, its success rate varies greatly, ranging from 25 to 100%, and it has the risk of damaging crossing vessels and iatrogenic ureteral injury (12–16). Balloon dilatation is often preferred as the initial procedure to salvage the recurrent UPJO, and the timing of surgery was not specified. It is believed that a narrow segment obstruction is probable to resolve with endopyelotomy or balloon dilatation where patients with the redundant pelvis (17, 18). However, for patients with kinking ureter, scar tissues, and multiple site narrowings, redo pyeloplasty is the most effective way to relieve obstruction and ensure urinary tract patency. Generally, ROP is the most commonly used procedure by surgeons in patients with recurrent UPJO. With the popularization of minimally invasive surgery, RLP is gradually being used for secondary pyeloplasty in some laparoscopically experienced centers. Small case series of RLP have demonstrated favorable success rates and outcomes (19, 20). However, few studies directly compared the clinical outcomes of RLP with those of PLP and ROP based on a large cohort.

In the present study, ten patients with recurrent UPJO were managed through RLP. Seven patients had previously undergone LP, and three had previously undergone OP. The interval between primary and redo pyeloplasty was 7.50 [6.25; 14.8] months. In our RLP series, scar tissues and adhesions were the two main causes of re-obstruction. There were five cases of scar tissues, two cases of adhesions, and one case each of stenotic UPJ area, crossing vessels, and UPJ polyps. According to our clinical observations, the extent of adhesions and fibrosis varies widely between patients, which may relate to the patient's healing factors and the technical difficulty in the primary operation. A poorly positioned anastomotic stoma may result in higher tension and increase the risk of recurrent UPJO (21). Besides, using thermal energy increase the risk of scar formation, which may cause UPJ re-obstruction (22). The median operative time of RLP was 158 [120; 197] min, which is shorter than previous studies (6, 23). Four patients difficult to insert a DJ stent during the operation, and a nephrostomy tube plus an external ureteral stent were indwelled as the alternative drainage method, but no ureterovesical junction obstruction was observed during the follow-up. Post-operatively, one patient had a paralytic ileus resolved after fasting and decompression with a gastrointestinal tube (Clavien II). All patients demonstrated an improvement in hydronephrosis without failure.

To prove the applicability of the RLP, we compared patients who were managed with RLP and patients who underwent PLP. We found significant differences between the two groups regarding age, weight, pre-operative APD, P/C ratio, operation time, and post-operative LOS. The pre-operative APD and P/C ratio were significantly higher in the RLP group, indicating that those kidneys that have experienced UPJO are less resistant to re-obstruction. However, the post-operative P/C value was comparable between the RLP group and PLP group, and the mean pre-operative and post-operative changes in P/C ratio were significantly greater in the RLP group than in the PLP group, showing a good effect of RLP in relieving re-obstruction. The operation time was significantly longer in the RLP group compared with the PLP group for secondary UPJO (158 vs. 110 min, *P* = 0.015). This is due to the need for more time to loosen the peripelvic and periureteral fibrosis, achieve the appropriate anatomical plane, and reposition the kidneys for tension-free anastomosis, especially in children with limited abdominal manipulation space. Post-operatively, the complication rates were 30.0% (3/10) and 14.1% (58/412) in the RLP and PLP groups, and the failure rates were none and 2.9% (12/412) in the RLP and PLP groups. The clinical outcomes were no significant difference between the two groups. However, younger age, lower weight, and worse severity of hydronephrosis have been confirmed and are associated with recurrent UPJO (24, 25). These confounding factors could have introduced bias and possibly influenced the results. Therefore, we used the PSM method to balance the pre-operative characteristics in RLP and PLP groups. After matching age, weight, surgeon experience, pre-operative APD, cortical thickness, and P/C ratio, the results remained consistent.

The matched comparison was also used between the RLP and ROP groups. After balancing the confounding factors (age and weight) by the PSM method, there were significant differences in intraoperative drainage methods and post-operative LOS. The operation time of the RLP group is longer than the ROP group, but the difference was not significant (158 min in the RLP group and 133 min in the ROP group, *P* = 0.244). Previously, Abdel-Karim et al. (23) reported a significantly longer operation time in RLP compared with ROP in a 39 case series. In our opinion, although the operation time of RLP is longer than ROP's, the operation's anatomy is clearer and easier than open surgery because of the magnification effect of laparoscopy (Figures 1B,C). The advantages of a clearer surgical view, sufficient exposure, and clear anatomical landmark position are more favorable to define the etiology and probable location of the re-obstruction. In the present study, the post-operative LOS was significantly shorter in the RLP group than in the ROP group (6 vs. 10 days, *P* = 0.003). This may relate to the different drainage methods. In the ROP group, the nephrostomy tube was removed in accordance with the methylene blue study before discharge, which was usually 10–14 days after surgery, and cystoscopic removal of the DJ stent was done under general anesthesia at 4–6 weeks post-operatively. The different drainage methods contributed to the different LOS, which could explain the difference of LOS between the RLP and the ROP group. Based on our clinical observations, children who undergo RLP tend to recover more quickly after surgery than those who undergo ROP, reflecting LP surgery's advantages for patients with less trauma and quicker post-operative recovery. Our findings are confirmed by those reported by Abdel-Karim et al. (23). Similarly, Piaggio et al. (19) reported a shorter mean hospitalization in RLP cases compared with ROP cases. Besides, although the post-operative complication rate in the RLP group was higher than that in the ROP group (30.0 vs. 20.9%), there was no significant difference, and there was a failure case in the ROP group. Compared with ROP, RLP has the advantages of a clearer surgical view, sufficient exposure, clearer anatomical landmark position, and minor trauma, with a comparable clinical outcome.

It is well-recognized that managing recurrent UPJO is technically challenging due to the extensively fibrotic tissue (26, 27). As the referral center for complex and challenging UPJO in China, we have accumulated experience with pyeloplasties in challenging scenarios. We believe that elucidating the cause of restenosis and identifying the site of obstruction is critical to the success of the redo pyeloplasty. As mentioned above, the use of thermal energy increases the risk of scar formation in the primary pyeloplasty, which may cause UPJ re-obstruction (22). Besides, if pathologically altered ureteral tissues, such as narrow segments or polyps, are left during primary pyeloplasty, re-obstruction is prone to occur because the cause of the obstruction is not completely relieved. In particular, cases with long segments and multiple ureteral strictures are easily missed. In the present study, one RLP case of intraoperative exploration showed the UPJ area was still stenotic, and one RLP case showed polyps. Missing crossing vessels is one of the most common causes of re-obstruction (28). In our RLP series, one patient showed a crossing vessel during secondary management. Hence, imaging data should be carefully analyzed before the primary surgery, and careful exploration should be done during surgery to avoid omissions. Al-Hazmi et al. (6) suggested magnetic resonance urography as a pre-operative anatomical and functional imaging study to assess the anatomy of the renal pelvis and evaluate the split renal function.

The advantage of RLP is observing the ureteral and UPJ area under direct vision during exploration. The operative field of vision under laparoscopy was broader, showing the anatomical structures more clearly than in open surgery. During the operation, we can look for the restenosis site from the distal ureter to the UPJ. When we performed RLP in those patients with prior ureterocalicostomy, the nephrostomy tube can assist in locating the renal pelvis, further elucidating the cause of restenosis and identifying the site of obstruction. A successful redo pyeloplasty relies on a spacious and unobstructed, watertight, and tension-free anastomotic segment. Precautions should be taken to preserve the periureteral sheath that contains the blood supply to the ureter, clean, and fine fashioning of ureteral and pelvic flaps should be carried out. Moreover, effective anatomical reduction and unobstructed drainage during the redo pyeloplasty are essential to prevent restenosis caused by inflammatory adhesions of connective tissue around the pelvis and ureter that compress the ureter or anastomosis. In addition, the experience of the surgeon in using LP may reduce the operation time and failure rate. Early in the learning curve, some surgeons may experience restenosis in patients due to unskilled suturing techniques, poor post-operative drainage leading to recurrent urinary tract infections, and inflammatory proliferation due to urine leakage (29).

There are several limitations worth noting. On the one hand, it is a retrospective study, and there might have some potential bias. On the other hand, given the low incidence of recurrent UPJO, the sample size of this study was small. A larger cohort would be necessary to expose small differences between RLP, PLP, and ROP. Although the good clinical outcomes in the present study support the RLP, enthusiasm for the technique must be tempered because of the small sample size and lack of a cost-effectiveness study. Additional randomized prospective studies, including larger sample sizes and longer follow-up periods are needed in the future.

## Conclusions

Our retrospective study demonstrated that RLP performed as well as PLP except for a longer operation time. Compared with ROP, RLP has the advantages of a clearer surgical view, sufficient exposure, clearer anatomical landmark position, and minor trauma, with a comparable clinical outcome. On experienced hands, RLP for recurrent UPJO after is a safe and effective procedure and should be considered an excellent alternative to the more commonly recommended ROP in select patients.

## Data availability statement

The raw data supporting the conclusions of this article will be made available by the authors, without undue reservation.

## Ethics statement

The Ethics Committee approved this retrospective study of Beijing Children's Hospital, Capital Medical University, National Center for Children's Health [IEC-C-006-A04-V.06, (2022)-E-030-R]. Written informed consent from the participants' legal guardian/next of kin was not required to participate in this study in accordance with the national legislation and the institutional requirements.

## Author contributions

JL, YY, and ZL developed the project, collected and analyzed the data, and wrote the manuscript. ZY, XW, and SF collected the data. PL, HS, and WZ participated in the operation and revised the article. All authors contributed to the article and approved the submitted version.

## Conflict of interest

The authors declare that the research was conducted in the absence of any commercial or financial relationships that could be construed as a potential conflict of interest.

## Publisher's note

All claims expressed in this article are solely those of the authors and do not necessarily represent those of their affiliated organizations, or those of the publisher, the editors and the reviewers. Any product that may be evaluated in this article, or claim that may be made by its manufacturer, is not guaranteed or endorsed by the publisher.

## Abbreviations

UPJO: ureteropelvic junction obstruction
PLP: primary laparoscopic pyeloplasty
RLP: redo laparoscopic pyeloplasty
ROP: redo open pyeloplasty
PSM: Propensity score matching
APD: anteroposterior diameter
P/C ratio: APD/cortical thickness
PI-APD: percentage of improvement of APD
LOS: length of stay
DJ: Double-J.
